# Association between daily sitting time and kidney stones based on the National Health and Nutrition Examination Survey (NHANES) 2007–2016: a cross-sectional study

**DOI:** 10.1097/JS9.0000000000001560

**Published:** 2024-05-20

**Authors:** Ya Li, Xingpeng Di, Mengzhu Liu, Jingwen Wei, Tianyue Li, Banghua Liao

**Affiliations:** Department of Urology and Institute of Urology (Laboratory of Reconstructive Urology), West China Hospital, Sichuan University, Chengdu, Sichuan, People’s Republic of China

**Keywords:** daily sitting time, kidney stone, National Health and Nutrition Examination Survey (NHANES), vigorous recreational activity

## Abstract

**Background:**

Kidney stones are among the most common urological conditions affecting ~9% of the world population. Although some unhealthy diets and unhealthy lifestyles are reportedly risk factors for kidney stone, the association between daily sitting time and kidney stone has not been explored.

**Materials and Methods:**

This large-scale, cross-sectional study was conducted using data from the National Health and Nutrition Examination Survey (NHANES) database 2007–2016. Kidney stone history and daily sitting time were retrieved from the questionnaire and 24 hour (h) recall interviews. Logistic regression and subgroup analysis were conducted to investigate the association. The analysis was further stratified by vigorous recreational activity.

**Results:**

A total of 19 188 participants aged ≥20 years with complete information were included in this study. The overall prevalence of kidney stone was 9.6%. Among participants without vigorous recreational activity, a trend towards an increasing prevalence of kidney stone was observed with increased daily sitting time. However, the trend was not observed in individuals who participated in vigorous recreational activity, as they experienced a decreased risk of kidney stone despite having a daily sitting time of 6–8 h (crude model OR=0.659, 95% CI: 0.457–0.950, *P*=0.028), indicating that vigorous recreational activity may partially attenuate the detrimental effect of prolonged sitting time.

**Conclusion:**

Our study revealed an increasing trend of prevalence of kidney stone with increased daily sitting time among the population not performing vigorous recreational activity despite the difference was nonsignificant. Vigorous recreational activity may modify the association between daily sitting time and kidney stone. More prospective cohort studies are warranted to further examine this association.

## Introduction

Highlights

- Over 30% of the world population of 15 years or older are physically inactive and this unhealthy lifestyle attributes to nearly 3.2 million deaths annually.
- The risk of kidney stone increases with increasing daily sitting time among participants without vigorous recreational activity.
- Vigorous recreational activity may partially modify the association between daily sitting time and kidney stone.

Kidney stone is one of the most prevalent urological conditions, with a prevalence rate of ~9% in the adult population worldwide, and the incidence of kidney stone continues to increase^1–3^. Minimally invasive surgeries and extracorporeal shockwave lithotripsy are effective methods for removing the stones, but the recurrence rate is still high up to 50%^4^. Hydronephrosis caused by kidney stones can impair renal function and lead to kidney dysfunction, which impact both life quality and expectancy of patients^5^. The annual health care expenditure for kidney stone was reported to exceed 2 billion dollars in the United States (US), which is an enormous burden for the health care system^6^. Therefore, elucidating the risk factors and etiology of kidney stone is vital for comprehensive disease management.

Sedentary behavior pertains to any awake activity performed while sitting, reclining, or lying down, with an energy expenditure of no more than 1.5 metabolic equivalents (METs)^7^. Sedentary behavior is considered a risk factor for various diseases, such as diabetes mellitus (DM), obesity, cardiovascular diseases, cancer, and dementia^8,9^. Meanwhile, prolonged sitting was associated with increased insulin demand and higher postprandial glucose in a randomized crossover study, while breaking up prolonged sitting time was associated with increased expression of anti-inflammatory and antioxidative molecules^10,11^. Since 2012, physical inactivity has been considered a pandemic^12^, and health care systems spent 53.8 billion in 2013 as a result of physical inactivity worldwide^13^. The WHO 2020 Global Guidelines on Physical Activity and Sedentary Behavior strongly recommended that people of all ages minimize sedentary time and spend more time on physical activities to gain benefits^14^.

Recent epidemiological surveys and clinical research have indicated a high comorbidity rate of kidney stone with systemic diseases such as DM, obesity, hypertension (HTN), and inflammatory bowel disease^15,16^. Metabolic syndrome, high BMI, low fluid intake and certain diets are risk factors for kidney stone^17,18^. Since unhealthy lifestyles are commonly recognized as important factors in the development of diseases such as DM and obesity, we should not neglect their potential impact on kidney stone. Although sedentary behaviors are associated with several risk factors for urinary stone, such as high BMI and inadequate physical activity, direct evaluations on the potential impact of sedentary behavior on the occurrence of urinary stone are scarce.

The National Health and Nutrition Examination Survey (NHANES) is a nationwide continuous program that conducted to evaluate the health and nutritional status of the population in the US. The survey covers a wide range of aspects related to human health, including demographic, socioeconomic, dietary, and health-related questions, interviews, examinations, and laboratory tests. In this study, we intended to explore the association between sitting time and kidney stone in US adults based on data retrieved from the NHANES and to provide guidance for the prevention of kidney stones.

## Materials and methods

### Study population

**Figure 1:**
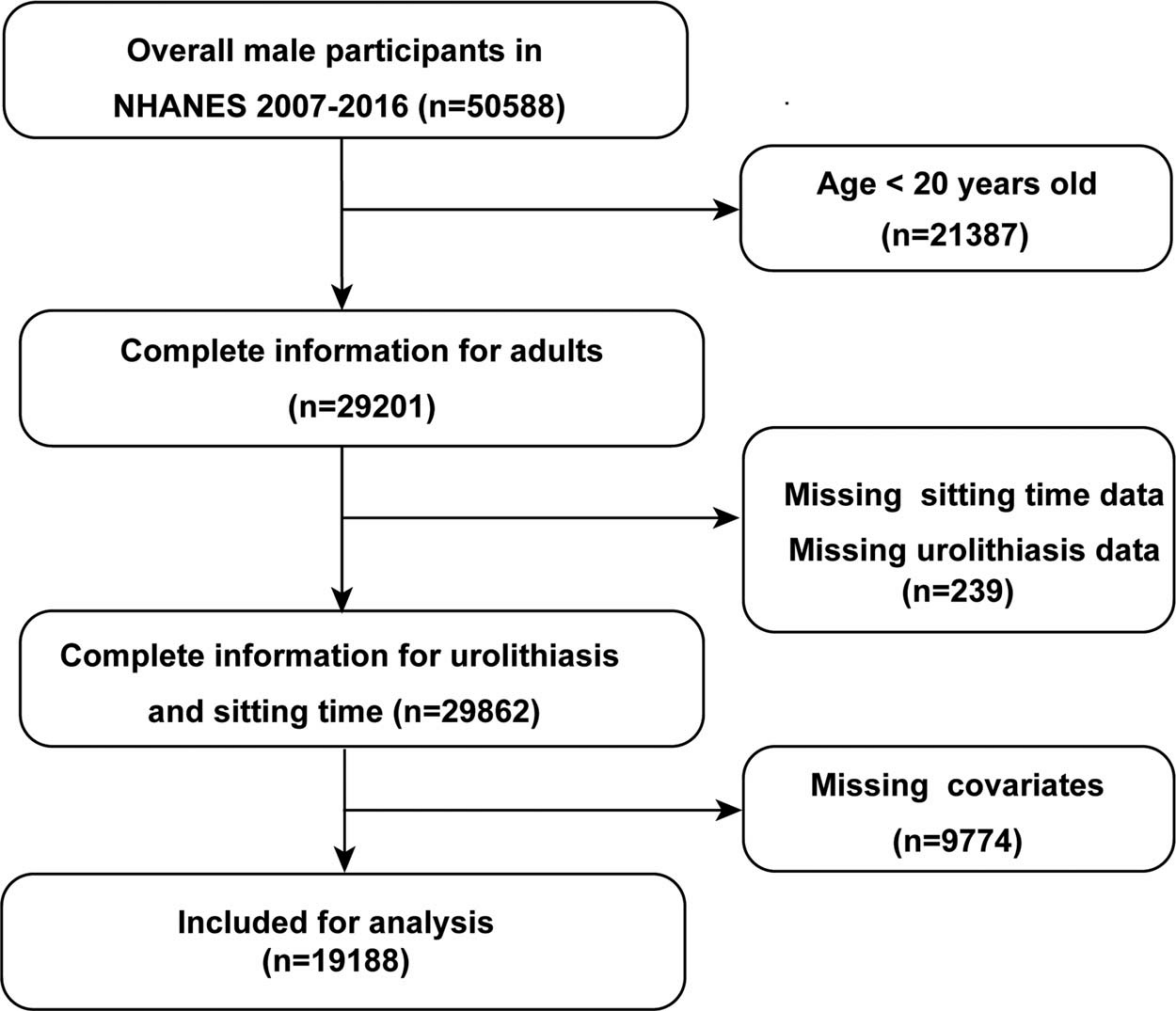
Flow diagram of participant screening. NHANES, National Health and Nutrition Examination Survey.

The NHANES dataset is an ongoing, nationwide, 2-year cycled, and cross-sectional survey that collects demographic, socioeconomic, nutritional, and health-related data from the US population. It combines interviews, physical examinations, and laboratory tests to assess the nation’s nutritional and health conditions. Five cycles of data in the NHANES database from 2007 to 2016 were utilized in this study. Participants who met the following inclusion criteria were included: (1) age ≥20 years; (2) with complete data on daily sitting time and kidney stone; (3) with data on vigorous recreational activity and other covariates (Fig. 1). Notably, the ethical review board of the National Center for Health Statistics granted approval to the NHANES protocols, and this study was exempted from ethical approval. All participants in this study signed informed consent forms.

### Sitting time

Daily sitting time was the exposure variable in our study, measured by the following question: ‘How much time do you usually spend sitting (or reclining) on a typical day?’ This variable referred to the number of waking hours spent ‘sitting or reclining at work, at home, or at school, including time spent sitting at a desk, sitting with friends, traveling in a car, bus, or train, reading, playing cards, watching television, or using a computer’. Daily sitting time was categorized into four groups^9,10^: less than 4 hours (h) per day, 4–6 h per day, 6–8 h per day, and 8 h or more per day. A daily sitting time of less than 4 h per day was defined as the reference in the subsequent analysis.

### Kidney stone history

Kidney stone history was identified from the Kidney Conditions-Urology survey in the questionnaire data. The survey on kidney stones was conducted by trained interviewers at home, and a computer-assisted personal interview (CAPI) system was utilized. The participants were asked ‘Have you ever had a kidney stone?’, and if the answer was ‘Yes’, the participant was considered to have the history of kidney stones.

### Covariates

Several additional covariates were collected and used for adjustment in this study to reduce potential bias based on previous studies^19–21^. In adjusted Model 1, four sociodemographic characteristics, namely, age, race, education level, and the family income-to-poverty ratio (defined as income relative to the poverty threshold), were considered. Model 2 was additionally adjusted for the following covariates: lifestyle characteristics such as smoking history, alcohol consumption history, moderate recreational activity status, and health status covariates such as BMI, DM, HTN, and coronary heart disease. A comprehensive description of these covariates is presented in Supplementary Table 1 (Supplemental Digital Content 1, http://links.lww.com/JS9/C577). In addition, moderate recreational activity refers to sports, fitness, or recreational activities of moderate intensity that result in a slight increase in breathing or heart rate for a minimum of 10 min a week, such as brisk walking, bicycling, swimming, golf, etc. As higher physical activity is a potential protective factor against kidney stones and the prevalence of kidney stone decreases as physical activity increases within a certain range^22,23^, a stratified logistic regression analysis based on whether participants engaged in vigorous recreational activity was constructed to better assess the association between daily sitting time and kidney stone prevalence. Vigorous recreational activity refers to continuous sports, fitness, or recreational activities of vigorous intensity that resulted in a substantial increase in breathing or heart rate for at least 10 min per week, such as running and basketball.

### Statistical analysis

We followed Centers for Disease Control and Prevention (CDC) guidelines for all the statistical analyses and applied appropriate sample weights for the enrollment of participants. Baseline characteristics for the enrolled population were described and stratified by different daily sitting time groups. The mean±SD was used to summarize continuous variables, while percentage was used to summarize categorical variables. Survey-weighted Analysis of Variance (ANOVA) was employed to assess differences between daily sitting time groups for continuous variables, and the survey-weighted *χ* ^2^ test was utilized for categorical variables. To analyze the association between daily sitting time and kidney stone, the crude model and adjusted models were applied in the logistic regression analysis: no adjustment was performed in the crude model; Model 1 was adjusted for age, race, education level, and family income-to-poverty ratio; and Model 2 was additionally adjusted for age, race, education level, family income-to-poverty ratio, BMI, smoking history, alcohol consumption history, DM, HTN, coronary heart disease, and moderate recreational activity. The strength of the association was estimated by odds ratios (ORs) and associated 95% Confidential intervals. To test for interaction effects between covariates and daily sitting time and assess the robustness of the results, we further performed interactive effect analysis stratified logistic regression analysis to identify variables that affect the association between sitting time and kidney stone in the vigorous recreational activity subgroup. The differences in kidney stone prevalence between individuals with and without vigorous recreational activity within the different daily sitting time groups were compared by Fisher’s exact test. Analyses were performed with *R* software (http://www.R-project.org; The R Foundation) and EmpowerStats version 4.0 (http://www.empowerstats.com, X&Y Solutions, Inc.). *P*<0.05 (two-sided) was considered as a significance.

This study was conducted following the guideline of strengthening the reporting of cohort, cross-sectional, and case–control studies in surgery (STROCSS 2021)^24^ (Supplemental Digital Content 2, http://links.lww.com/JS9/C578).

## Results

### Population characteristics

From 2007 to 2016, a total of 50 588 individuals were initially recruited in the survey. A total of 19 188 participants aged ≥20 years with complete information were included in our study for further analysis (Fig. 1). A total of 10 235 (53.3%) of the participants were females, and the other 8953 (46.7%) were males. A total of 8936 out of 19 188 (46.6%) participants were non-Hispanic white, 3936 (20.5%) were non-Hispanic black, 2695 (14.0%) were Mexican American, 1896 (9.9%) were other Hispanic, and 1725 (9.0%) were participants of other races, including American Indian/Native Alaskan/Pacific Islander, Asian, and multiracial. More than a quarter of the participants reported that they sat for 6–8 h per day, while those who sat for more than 8 h accounted for 21.0% of the total population. Mexicans were more likely to sit for less than 4 h per day (1127/2695, 41.8%), while participants of other races were the least likely to sit for less than 4 h per day (311/1725, 18.0%). In addition, participants who sat longer were more likely to have higher levels of education, a higher family income-to-poverty ratio, a higher BMI, nonsmokers status, and a lower likelihood of engaging in vigorous recreational activity. Daily sitting time also differed between individuals with different health statuses. The participants who sat for 6–8 h were older (50.6±18.0) and had a higher prevalence of DM (20.1%), HTN (46.3%), and coronary heart disease (5.0%). Moreover, daily sitting time did not differ between participants with or without moderate recreational activities.

Among all participants, 9.6% of the population had kidney stones, and the percentages of patients with kidney stones per group were 8.7%, 9.7%, 10.0%, and 10.2% for those sitting <4, 4–6, 6–8, and >8 h per day, respectively. Despite the trend of a higher prevalence of kidney stone in participants who sat for longer hours, no significant difference was observed between the groups (*P*=0.457). The detailed baseline characteristics of the participants classified by quartile of sitting time per day are presented in Table 1.

**Table 1:** Characteristics of participants by categories of sitting time per day: NHANES 2007–2016, weighted.

	Sitting time/day (h)				
Characteristics	<4	4 to <6	6 to 8	>8	*P*
Number (n)	4899	4575	5688	4026	
Age	47.4±16.3	49.5±17.7	50.6±18.0	48.3±17.1	<0.0001
Sex (n/%)					0.601
Female	2682 (54.7%)	2502 (54.7%)	2984 (52.5%)	2067 (51.3%)	
Male	2217 (45.3%)	2073 (45.3%)	2704 (47.5%)	1959 (48.7%)	
Race (*n*/%)					<0.0001
Mexican American	1127 (23.0%)	650 (14.2%)	601 (10.6%)	317 (7.9%)	
Non-Hispanic Black	920 (18.8%)	937 (20.5%)	1220 (21.4%)	859 (21.3%)	
Non-Hispanic White	1894 (38.7%)	2155 (47.1%)	2776 (48.8%)	2111 (52.4%)	
Other Hispanic	647 (13.2%)	464 (10.1%)	510 (9.0%)	275 (6.8%)	
Other race	311 (6.3%)	369 (8.1%)	581 (10.2%)	464 (11.5%)	
Education level (*n*/%)					<0.0001
Lower than 12th grade	1619 (33.0%)	1094 (23.9%)	1095 (19.3%)	494 (12.3%)	
High school grade	1242 (25.4%)	1125 (24.6%)	1252 (22.0%)	738 (18.3%)	
College grade	2038 (41.6%)	2356 (51.5%)	3341 (58.7%)	2794 (69.4%)	
Family income-to-poverty ratio (*n*/%)					<0.0001
<1.3	1839 (37.5%)	1440 (31.5%)	1729 (30.4%)	886 (22.0%)	
>=1.3, <3.5	1978 (40.4%)	1777 (38.8%)	2051 (36.1%)	1292 (32.1%)	
>=3.5	1082 (22.1%)	1358 (29.7%)	1908 (33.5%)	1848 (45.9%)	
BMI (kg/m^2^, *n*/%)					<0.0001
<=20	200 (4.1%)	224 (4.9%)	264 (4.6%)	164 (4.1%)	
>20, <=25	1339 (27.3%)	1140 (24.9%)	1358 (23.9%)	940 (23.3%)	
>25, <=30	1732 (35.4%)	1592 (34.8%)	1787 (31.4%)	1230 (30.6%)	
>30	1628 (33.2%)	1619 (35.4%)	2279 (40.1%)	1692 (42.0%)	
Smoking history (*n*/%)					0.008
Non-smoker	2431 (49.6%)	2193 (47.9%)	2750 (48.3%)	2062 (51.2%)	
Smoker	2468 (50.4%)	2382 (52.1%)	2938 (51.7%)	1964 (48.8%)	
Moderate activity (*n*/%)					0.245
No	2918 (59.6%)	2554 (55.8%)	3351 (58.9%)	2295 (57.0%)	
Yes	1981 (40.4%)	2021 (44.2%)	2337 (41.1%)	1731 (43.0%)	
Vigorous activity (*n*/%)					0.01
No	3788 (77.3%)	3528 (77.1%)	4440 (78.1%)	2990 (74.3%)	
Yes	1111 (22.7%)	1047 (22.9%)	1248 (21.9%)	1036 (25.7%)	
Alcohol drinking history (drinks/week, *n*/%)					0.011
<1	3182 (65.0%)	2853 (62.4%)	3606 (63.4%)	2402 (59.7%)	
1–3	1228 (25.1%)	1191 (26.0%)	1448 (25.5%)	1155 (28.7%)	
>= 4	489 (10.0%)	531 (11.6%)	634 (11.1%)	469 (11.6%)	
Diabetes mellitus (*n*/%)					<0.0001
No	4133 (84.4%)	3774 (82.5%)	4547 (79.9%)	3291 (81.7%)	
Yes	766 (15.6%)	801 (17.5%)	1141 (20.1%)	735 (18.3%)	
Hypertension (*n*/%)					<0.0001
No	3093 (63.1%)	2616 (57.2%)	3055 (53.7%)	2297 (57.1%)	
Yes	1806 (36.9%)	1959 (42.8%)	2633 (46.3%)	1729 (42.9%)	
Coronary heart disease (*n*/%)					0.0001
No	4761 (97.2%)	4398 (96.1%)	5402 (95.0%)	3837 (95.3%)	
Yes	138 (2.8%)	177 (3.9%)	286 (5.0%)	189 (4.7%)	
Kidney stone history (*n*/%)					0.457
No	4472 (91.3%)	4132 (90.3%)	5121 (90.0%)	3614 (89.8%)	
Yes	427 (8.7%)	443 (9.7%)	567 (10.0%)	412 (10.2%)	

Mean±SD for continuous variables, *P*<0.05 presents significant difference.

DM, diabetes mellitus; h, hour; NHANES, National Health and Nutrition Examination Survey.

### Logistic regression analysis of kidney stone risk

**Figure 2:**
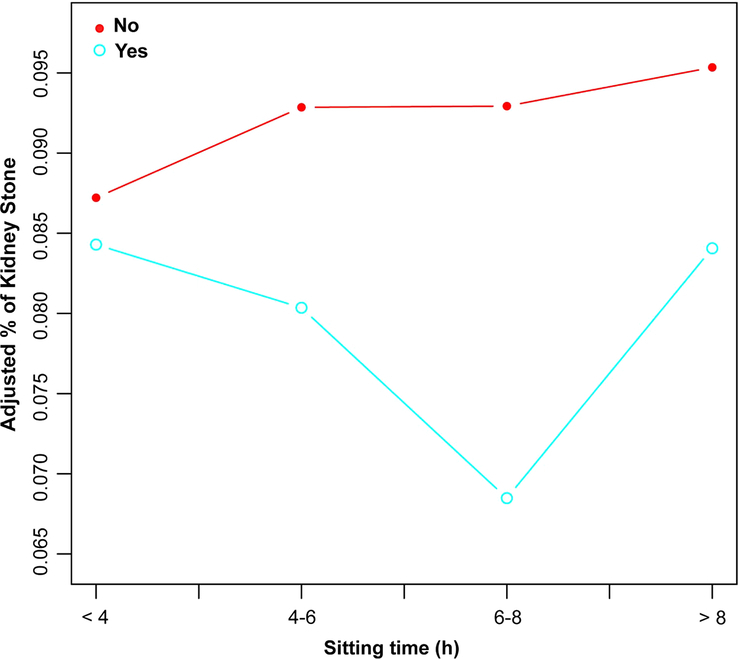
Adjusted prevalence of kidney stone among participants with different daily sitting time. Red indicates participants without vigorous activity. Blue indicates participants with vigorous activity.

We further examined the association between daily sitting time and kidney stones by weighted logistic regression model, with those sitting for less than 4 h per day serving as the reference group. Among participants with vigorous recreational activity, univariate logistic regression analysis indicated that the risk of kidney stone was 34.1% lower in those who sat for 6–8 h per day (OR=0.659, 95% CI: 0.457–0.950, *P*=0.028) and 7% lower in those who sat for 4–6 h (OR=0.930, 95% CI: 0.601–1.437, *P*=0.744) than in those who sat for less than 4 h. The risk of kidney stone increased when patients sat longer than 8 h per day (OR=1.123, 95% CI: 0.753–1.675, *P*=0.571). Notably, the association of sitting for 6–8 h per day with kidney stone was still significant after adjustment for covariates in Model 1 (OR=0.640, 95% CI: 0.420–0.976, *P*=0.042) and Model 2 (OR=0.606, 95% CI: 0.396–0.928, *P*=0.022). Interestingly, none of these associations between daily sitting time and kidney stone were significant in participants without vigorous recreational activity. Adjusting for covariates did not change the results (Table 2). Moreover, a possible trend towards an increased prevalence of kidney stone was observed with increased daily sitting time (Fig. 2). The adjusted prevalence of kidney stone with 95% Confidential intervals in different daily sitting time groups stratified by vigorous recreational activity status is provided in Supplementary Figure 1 (Supplemental Digital Content 1, http://links.lww.com/JS9/C577).

**Table 2:** Univariate and multivariate analyses by activity-stratified logistic regression model, weighted.

	Sitting time/day (h)			
Vigorous activity	<4 (OR, 95% CI), *P*	4 to <6 (OR, 95% CI), *P*	6 to 8 (OR, 95% CI), *P*	>8 (OR, 95% CI), *P*
Yes				
Crude model^a^	Reference	0.930 (0.601–1.437), 0.744	0.659 (0.457–0.950), 0.028	1.123 (0.753–1.675), 0.571
Model 1^b^	Reference	0.924 (0.582–1.469), 0.741	0.640 (0.420–0.976), 0.042	1.166 (0.708–1.919), 0.548
Model 2^c^	Reference	0.908 (0.571–1.443), 0.677	0.606 (0.396–0.928), 0.022	1.125 (0.677–1.870), 0.644
No				
Crude model	Reference	1.025 (0.838–1.254), 0.808	1.146 (0.939–1.399), 0.183	1.166 (0.975–1.393), 0.096
Model 1	Reference	0.975 (0.797–1.193), 0.804	1.069 (0.876–1.303), 0.515	1.122 (0.936–1.346), 0.218
Model 2	Reference	0.941 (0.763–1.161), 0.574	0.965 (0.787–1.183), 0.732	0.978 (0.810–1.180), 0.814

a Crude model1: adjusted for none.

b Model1: adjusted for age, race, education level, and family income ratio.

c Model2: adjusted for age, race, education level, family income-to-poverty ratio, BMI, smoking history, alcohol drinking history, DM, hypertension, coronary heart disease, and moderate activity. *P*<0.05 presents significant difference.

DM, diabetes mellitus; h, hour; OR, odds ratio.

### Subgroup analysis

Stratified logistic regression analysis as well as interactive effect analysis were further performed among participants with vigorous recreational activity to identify variables that may modify the association between daily sitting time and kidney stone. Only smoking and comorbidity with coronary heart disease may influence the association between daily sitting time and kidney stone among participants with vigorous recreational activity (Table 3). Other covariates, such as age, sex, and race, did not impact kidney stone prevalence in the group sitting 6–8 h per day. In addition, in patients with coronary heart disease, the risk of kidney stone increased significantly with prolonged sitting time, while this association was not observed for those without coronary heart disease. Further analysis of the association between vigorous recreational activity and kidney stone indicated a lower prevalence of kidney stone in participants who performed vigorous recreational activity regardless of daily sitting time (Table 4).

**Table 3:** Stratified logistic regression analysis to identify variables that modify the correlation between sitting time and kidney stone in vigorous activity subgroup, weighted.

	Sitting time/day (h)^a^				
Characteristics	<4 (OR, 95% CI)	4 to <6 (OR, 95% CI)	6 to 8 (OR, 95% CI)	>8 (OR, 95% CI)	*P* for interaction
Age					0.937
20–39	Reference	1.00 (0.49–2.03)	0.81 (0.40–1.63)	1.37 (0.60–3.13)	
40–59	Reference	0.73 (0.36–1.50)	0.45 (0.23–0.88)	0.88 (0.42–1.85)	
60–80	Reference	1.22 (0.44–3.41)	0.70 (0.24–2.04)	1.34 (0.48–3.73)	
Sex (*n*/%)					0.841
Female	Reference	0.78 (0.36–1.67)	0.62 (0.27–1.44)	0.95 (0.43–2.10)	
Male	Reference	0.98 (0.60–1.61)	0.60 (0.39–0.92)	1.22 (0.67–2.22)	
Race (*n*/%)					0.006
Mexican American	Reference	0.43 (0.16–1.16)	1.45 (0.65–3.25)	1.10 (0.43–2.80)	
Non-Hispanic Black	Reference	0.44 (0.14–1.34)	0.59 (0.22–1.58)	0.65 (0.22–1.93)	
Non-Hispanic White	Reference	0.94 (0.55–1.58)	0.54 (0.33–0.88)	1.19 (0.67– 2.12)	
Other Hispanic	Reference	1.27 (0.39–4.15)	1.41 (0.52–3.81)	0.56 (0.15–2.09)	
Other race	Reference	1.36 (0.25–7.34)	0.55 (0.14–2.21)	0.78 (0.15–4.11)	
Education level (*n*/%)					0.556
Lower than 12th grade	Reference	1.80 (0.45–7.31)	1.27 (0.43–3.80)	0.53 (0.06–4.92)	
High school grade	Reference	0.91 (0.38–2.18)	0.65 (0.25–1.65)	1.26 (0.34–4.70)	
College grade	Reference	0.81 (0.49–1.34)	0.55 (0.33–0.90)	1.05 (0.63–1.73)	
Family income ratio (*n*/%)					0.848
<1.3	Reference	0.58 (0.19–1.71)	0.56 (0.18–1.79)	1.55 (0.39–6.16)	
>=1.3, <3.5	Reference	0.97 (0.50–1.87)	0.69 (0.33–1.41)	0.83 (0.32–2.14)	
>=3.5	Reference	0.96 (0.52–1.77)	0.58 (0.32–1.07)	1.22 (0.68–2.18)	
BMI (kg/m^2^,*n*/%)					0.219
<=20	Reference	0.46 (0.08–2.70)	0.15 (0.02–1.07)	0.52 (0.05–5.28)	
>20, <=25	Reference	0.82 (0.34–1.98)	0.82 (0.34–1.95)	0.91 (0.34–2.41)	
>25, <=30	Reference	0.55 (0.26–1.15)	0.50 (0.26–0.97)	1.27 (0.60–2.67)	
>30	Reference	1.95 (0.82–4.65)	0.70 (0.38–1.29)	1.29 (0.53–3.12)	
Smoking history (*n*/%)					0.035
Non-smoker	Reference	0.84 (0.45–1.57)	0.75 (0.43–1.32)	1.75 (0.94–3.25)	
Smoker	Reference	0.96 (0.51–1.82)	0.50 (0.27–0.90)	0.58 (0.29–1.18)	
Alcohol drinking history (drinks/week, *n*/%)					0.638
<1	Reference	0.76 (0.42–1.40)	0.43 (0.25–0.74)	0.99 (0.49–2.03)	
1-3	Reference	1.69 (0.63–4.53)	1.17 (0.51–2.68)	1.78 (0.74–4.28)	
>= 4	Reference	0.57 (0.15–2.19)	0.59 (0.12–2.85)	0.94 (0.26–3.45)	
Moderate activity (*n*/%)					0.436
No	Reference	1.39 (0.56–3.44)	0.90 (0.35–2.32)	2.01 (0.74–5.40)	
Yes	Reference	0.81 (0.50–1.30)	0.55 (0.35–0.86)	0.95 (0.61–1.47)	
Diabetes mellitus (*n*/%)					0.267
No	Reference	0.85 (0.53–1.37)	0.57 (0.36–0.90)	1.02 (0.61–1.71)	
Yes	Reference	2.27 (0.55–9.46)	1.44 (0.26–8.02)	3.59 (1.00–12.86)	
Hypertension (*n*/%)					0.975
No	Reference	0.85 (0.52–1.41)	0.58 (0.34–0.99)	1.09 (0.59–2.03)	
Yes	Reference	1.04 (0.47–2.29)	0.67 (0.31–1.45)	1.22 (0.57–2.57)	
Coronary heart disease (*n*/%)					0.011
No	Reference	0.87 (0.55–1.38)	0.57 (0.37–0.86)	1.08 (0.65–1.78)	
Yes	Reference	18.93 (1.56–229.63)	34.69 (3.04–396.13)	33.35 (1.92–580.53)	

a Fully adjusted model: adjusted for age, race, education level, family income-to-poverty ratio, BMI, smoking history, alcohol drinking history, DM, hypertension, coronary heart disease, and moderate activity. *P*<0.05 presents significant difference. All the models are not adjusted for the variable itself in each stratification.

**Table 4:** Comparison of prevalence of kidney stone between participants with vigorous recreational activity and those without.

		Kidney stone		
sitting time/day (h)	Vigorous recreational activity	Yes	No	*P*
<4	Yes	1.49%	21.19%	0.0036
	No	7.23%	70.10%	
4 to <6	Yes	1.51%	21.38%	<0.0001
	No	8.17%	68.94%	
6 to 8	Yes	1.21%	20.73%	<0.0001
	No	8.76%	69.30%	
>8	Yes	1.69%	24.04%	<0.0001
	No	8.54%	65.72%	

*P*<0.05 presents significant difference.

## Discussion

The WHO estimated that 31% of the world population aged 15 years or older is physically inactive, and this unhealthy lifestyle contributes to nearly 3.2 million deaths annually^25^. Most adults are reported to spend the majority of their daily waking hours sitting^26^, while the total sitting time has increased by almost 1 h per day among adults in the US over the past decade according to research based on NHANES survey data^27,28^. Moreover, the situation worsened during the COVID-19 pandemic, as restricted access to public exercise facilities were linked to an increase in sedentary behavior and a decrease in physical activity^29^.

Physical inactivity and sedentary behaviors have been recognized as important risk factors for many noncommunicable chronic diseases; however, research on their potential impact on the risk of kidney stone is relatively scarce^9,30^. In the current study, we assessed the association between daily sitting time and kidney stone based on the NHANES data. In general, our results showed an increasing trend in the adjusted prevalence of kidney stone with increased sitting time in the population without vigorous recreational activity, despite further logistic regression analysis revealing that the difference was nonsignificant. We enrolled in 19 188 participants in the analysis, which seemed to be adequate. However, due to the assessment of kidney stone was subjective and daily sitting time was based on rough estimates, the intragroup heterogeneity could be very significant. We suspect that a larger sample size may lead to a statistically significant result. Importantly, in the vigorous recreational activity group, the potential hazardous effect of sitting for 6–8 h daily on kidney stone was not observed, indicating that vigorous recreational activity may partly attenuate the hazardous effect of prolonged sitting time on kidney stone. However, we cannot ignore other factors that may cause these differences, such as daily water intake, diet, and occupation, since these data are difficult to retrieve from the database. Nevertheless, a lower prevalence of kidney stone was observed in association with vigorous recreational activity regardless of daily sitting time. In addition, our subgroup analysis indicated that among coronary heart disease patients, the risk of kidney stone still significantly increased with prolonged daily sitting time even with vigorous recreational activity. For these patients, limiting daily sitting time is vital for lowering kidney stone risk.

The link between physical activity and kidney stones has long been controversial. Sorensen MD *et al*.^31^ reported that even the lowest level of physical activity in women reduced the risk of kidney stone formation by 16%, independent of caloric intake and BMI, while other studies observed no independent association^32,33^. Nevertheless, a more recent dose-response analysis based on NHANES data reported a negative relationship between physical activity and kidney stone, with a plateau of physical activity benefits at ~2480 MET-min week-1^23^. In our study, the protective effect of vigorous recreational activity on kidney stones was robust. The potential underlying mechanism could be multifaceted. First, vigorous recreational activity is likely to promote sweating, which leads to increased loss of water and sodium. The feedback loop of the renin-angiotensin-aldosterone system inversely promotes water intake and reabsorption of the distal convoluted renal tubule and collecting duct^34^. Moreover, physical activity might promote bone calcium deposition and thus reduce urinary calcium excretion, which is related to the formation of calcium-containing stones^35,36^. On the other hand, physical activity can impact many known risk factors for kidney stone, and thus indirectly reduce the prevalence of kidney stone. Increasing physical activity is one of the fundamental approaches to managing metabolic syndrome, which is proven to be highly correlated with kidney stone^37^. In addition, physical activity can also shape the gut microbiota in various manners and has been considered to play a significant role in kidney stone formation in recent research^38^.

Prolonged sitting time is commonly considered synonymous with physical inactivity in many cases. However, physical activity cannot always attenuate or eliminate the detrimental association between increased sitting time and many diseases^39^. Some existing studies suggest that prolonged sitting time is detrimental to metabolic health, even in the presence of regular physical activity, suggesting a partially independent health effect of sitting apart from physical activity^40^. The association between sitting time and kidney stone has not been reported in previous studies, and the role of physical activity between them also remains unknown. Our study reported a quasi-significant association between daily sitting time and kidney stone in people without vigorous recreational activity, suggesting that prolonged daily sitting time may be detrimental for the physically inactive population. We suggest that people who are physically inactive stand up and walk around more often to reduce their risk of developing kidney stones. More importantly, since the detrimental effect of sedentary behavior on kidney stones was not observed in populations with vigorous recreational activity, we strongly recommend that people with any amount of daily sitting time engage in vigorous physical activity on a regular basis.

To the best of our knowledge, this is the first study to investigate the potential role of daily sitting time on kidney stone, and we found that vigorous recreational activity may modify the effect of daily sitting for 6–8 h on kidney stones in a protective manner. Nevertheless, our study has several limitations. Since our study is based on the NHANES database, the cross-sectional study design makes the causal relationship difficult to explain, and the generalizability of our results may be affected since only the US population was included in this database. In addition, exposure and outcome variables were self-reported, which may introduce self-report and recall bias into the analysis and omit potential asymptomatic kidney stone patients. Information on some risk factors for kidney stones, such as daily water intake, was not provided in the database; thus, our adjustment may not be comprehensive enough. Moreover, we ignored the duration and frequency of vigorous recreational activity for kidney stones in the stratified analysis, which may also lead to bias. The finding of a lower risk of kidney stone among participants who participated in vigorous recreational activity and sat for 6–8 h daily was interesting, but there is a lack of support from other data sources, and exploration of the underlying mechanism would be of great value. Prospective cohort studies with objective methods to record physical activity and sitting time are required in the future to further confirm the results and explore the potential mechanism involved. Furthermore, the effects of physical activity of different intensities and durations should be examined more precisely (such as the effects of different levels of METs), and the effects of different types of physical activity should also be assessed separately.

## Conclusion

Our study revealed an increasing trend in the prevalence of kidney stone with increased daily sitting time among participants who did not participate in vigorous recreational activity, although the related logistic regression analysis revealed nonsignificant results. Moreover, the prevalence of kidney stone decreased significantly among the population with vigorous recreational activity compared with those without, regardless of the daily sitting time group. In addition, taking vigorous recreational activity may even modify the association between daily sitting time and kidney stone that we observed in the population without vigorous recreational activity. Prospective cohort studies are warranted to further examine this association.

## Ethical approval

The ethical review board of the National Center for Health Statistics granted approval to the NHANES protocols, and ethical approval was exempted in this study.

## Consent

Every participant in this study signed the informed consent forms.

## Sources of funding

This work was supported by the Project of Science and Technology Department of Sichuan Province (Grant Number: 2023YFS0029), and National postdoctoral researcher program of China (Grant Number: GZC20231800).

## Author contribution

Y.L.: study design, data interpretation, and manuscript writing; X.D.: study concept, data analysis, and revision of the manuscript; M.L.: study design and data analysis; J.W. and T.L.: data analysis; B.L.: study concept and revision of the manuscript.

## Conflicts of interest disclosure

We declare that there are no conflicts of interests.

## Research registration unique identifying number (UIN)

Not required, as we used de-identified NHANES data.

## Guarantor

Banghua Liao.

## Data availability statement

Data are open access and can be downloaded at: https://wwwn.cdc.gov/Nchs/Nhanes/.

## Provenance and peer review

Not commissioned, externally peer-reviewed.

## Supplementary Material
