# Supplementary material for: Association between daily sitting time and kidney stones based on the National Health and Nutrition Examination Survey (NHANES) 2007–2016: a cross-sectional study
Source: Int J Surg. 2024 May 20;110(8):4624–32. doi: 10.1097/JS9.0000000000001560 (PMC11325893; doi:10.1097/JS9.0000000000001560)
Supplement: Supplementary file 1 [file js9-110-4624-s001.pdf]

Supplemental Digital Content (SDC)

Supplementary table 1. Detailed description and options of covariables in the NHANES database.

Covariables	Description in NHANES
**Age**	Age
**Race**	Race (Mexican American, Other Hispanic, Non-Hispanic Black, Non-Hispanic White, Other Race)
**Education level**	Education level (lower than 12th grade, high school grade, college grade)
**Family income-to-poverty ratio**	A ratio of family income to poverty (< 1.3, 1.3–3.5 and >3.5)
**BMI**	Body mass index (≤20.0 kg/m2, 20-25 kg/m2, 25-30 kg/m2 and >30.0 kg/m2)
**Smoking history**	Participants were asked whether they smoked at least 100 cigarettes in life (yes and no)
**Alcohol drinking history**	Patients were asked how often do they drink alcohol over past 12 months or how many days do they drink alcohol per week, month, year (drinks/week <1; 1-3; ≥4)
**DM**	Participants were diagnosed as DM if: doctor told you have diabetes, or glycohemoglobin HbA1c(%) >= 6.5, or fasting glucose (mmol/l) >= 7.0, or, random blood glucose (mmol/l) >= 11.1, or two-hour OGTT blood glucose (mmol/l) >= 11.1, or use of diabetes medication or insulin, or asked whether they had diabetes (yes and no)
**HTN**	Participants were diagnosed as HTN if their average blood pressure was above 140/90 mmHg. Average blood pressure was calculated by the following protocol: the diastolic reading with zero is not used to calculate the diastolic average. If all diastolic readings were zero, then the average would be zero. If only one blood pressure reading was obtained, that reading is the average. If there is more than one blood pressure reading, the first reading is always excluded from the average. Participants were asked whether they had high blood pressure (yes and no)
**Coronary heart disease**	Patients were asked whether they have been told they had coronary heart disease (yes and no)
**Moderate recreational activity**	Patients were asked does they work involve moderate-intensity activity that causes small increases in breathing or heart rate such as brisk walking or carrying light loads for at least 10 minutes continuously (yes and no)

Supplementary figure 1. Adjusted prevalence of kidney stone in different daily sitting time group with its 95% CI.

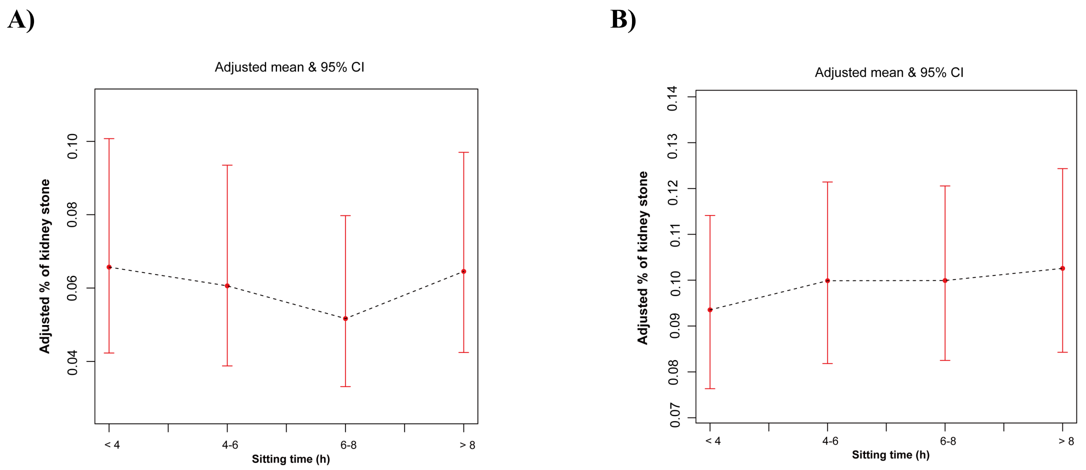

A) With vigorous recreational activity; B) Without vigorous recreational activity.
